# Dysregulated angiogenesis in B-chronic lymphocytic leukemia: Morphologic, immunohistochemical, and flow cytometric evidence

**DOI:** 10.1186/1746-1596-3-16

**Published:** 2008-04-18

**Authors:** John L Frater, Neil E Kay, Charles L Goolsby, Susan E Crawford, Gordon W Dewald, LoAnn C Peterson

**Affiliations:** 1 Department of Pathology, Northwestern University Feinberg School of Medicine, Chicago, USA; 2 Division of Hematology-Oncology, Department of Medicine, Mayo Clinic, Rochester, USA; 3 Cytogenetics Laboratory, Mayo Clinic, Rochester, USA

## Abstract

**Background:**

The extent of enhanced bone marrow angiogenesis in chronic lymphocytic leukemia (CLL) and relationship to proangiogenic factors and prognostic indicators is largely unexplored.

**Methods:**

To further investigate the role of angiogenesis in CLL by evaluating the topography and extent of angiogenesis in a group of CLL bone marrow biopsies, to study the expression of pro and antiangiogenic vascular factors in CLL cells to more precisely document the cell types producing these factors, and to evaluate the role, if any, of localized hypoxia in upregulation of angiogenesis in CLL We used immunohistochemistry (IHC) (n = 21 pts) with antibodies to CD3 and CD20, proangiogenic (VEGF, HIF-1a) and antiangiogenic (TSP-1) factors, and VEGF receptors -1 and -2 to examine pattern/extent of CLL marrow involvement, microvessel density (MVD), and angiogenic characteristics; flow cytometry (FC) was performed on 21 additional cases for VEGF and TSP-1.

**Results:**

CLL patients had higher MVD (23.8 vs 14.6, p~0.0002) compared to controls (n = 10). MVD was highest at the periphery of focal infiltrates, was not enhanced in proliferation centers, and was increased irrespective of the presence or absence of cytogenetic/immunophenotypic markers of aggressivity. By IHC, CLL cells were VEGF(+), HIF-1a (+), TSP-1(-), VEGFR-1(+), and VEGFR-2(+). By FC, CLL cells were 1.4–2.0-fold brighter for VEGF than T cells and were TSP-1(-).

**Conclusion:**

CLL demonstrates enhanced angiogenesis, with increased MVD, upregulated VEGF and downregulated TSP-1. Upregulation of HIF-1a in all CLL cases suggests localized tissue hypoxia as an important stimulant of microvessel proliferation. The presence of VEGF receptors on CLL cells implies an autocrine effect for VEGF. Differences in MVD did not correlate with traditional genetic/immunophenotypic markers of aggressiveness.

## Introduction

Angiogenesis, the branching of new microvessels from pre-existent larger blood vessels, is of major importance in normal embryogenesis and in physiologic processes such as ovulation and the menstrual cycle. Under normal conditions, an organ system is kept at a set point in which the pro- and antiangiogenic molecules are in a state of equilibrium. In neoplasia, the set point may become unbalanced in favor of proangiogenic molecules. This "angiogenic switch" [1] favors the production of new microvessels, thus facilitating tumor growth beyond 1–2 mm diameter, and metastasis of the malignant clone. A number of molecules, including vascular endothelial growth factor (VEGF), basic fibroblast growth factor (bFGF), and hypoxia-inducible factor 1α (HIF-1α) have been identified as positive regulators of angiogenesis. These are kept in balance by negative regulators of angiogenesis including thrombospondin-1 (TSP-1) [2] and interferon β (IFNβ) [3].

Pathologic angiogenesis was recognized to be important in the progression of solid tumors over 30 years ago [4]. More recently, abnormal angiogenesis has been identified in a number of hematologic malignancies. Although studies are limited, an increasing body of evidence supports the existence of increased tissue site angiogenesis in chronic lymphocytic leukemia (CLL). An increase in microvessel density, an index of angiogenic activity defined by the number of microvessels per microscopic high power field, was noted in CLL bone marrows by Kini et al; the degree of angiogenesis correlated with Rai stage [5]. Increased microvessel density has also been noted in other sites including lymph nodes involved by CLL [6]. Vascular factors relevant in angiogenesis including VEGF and bFGF have been reported in increased levels in serum and urine of some CLL patients [5,7-9]. Kay et al report increased VEGF and bFGF in the supernatant of CLL cells grown in vitro and upregulation of mRNA encoding VEGF and its receptors and bFGF, suggesting that angiogenic factors are important in the biology of the malignant B-cell clone [10]. In addition to its role in angiogenesis, bFGF appears to upregulate BCL-2 expression in CLL [11]. In vitro evidence suggests that in a subset of CLL cases TSP-1 is expressed by a subset of CLL cells [12]. Microvessel density correlates with stage and progression free survival in CLL [5] and increased expression of VEGF receptors and bFGF correlate with clinical stage [7,13]. However, the extent to which increased angiogenesis correlates with known genetic and immunophenotypic prognostic factors in CLL is not known. In addition, similar studies to those reviewed above have not been performed in the bone marrow of CLL patients.

In hypoxic conditions, normal tissues express HIF-1α, which upregulates expression of VEGF and other factors, resulting in increased angiogenesis and increased oxygen delivery to tissues [14]. In a deregulated system, abnormally increased HIF-1α may likewise increase angiogenesis and it has been demonstrated that hypoxia exists in normal marrows [15]. CLL cells have been demonstrated to have the ability to produce significant levels of VEGF under hypoxic conditions [12]. Also, cells from CLL-derived lines have been shown to secrete HIF-1α [10]. Thus it is likely that hypoxia plays a role in CLL B-cell secretion of VEGF.

The aforementioned studies suggest that angiogenesis may be of major importance in CLL. A direct assessment of pro- and antiangiogenic molecules, their receptors, and the level of HIF-1α in relationship to the environment where CLL is believed to originate, the bone marrow, would add further credence to the argument that angiogenesis is of importance in the pathophysiology of CLL. Moreover, it would be possible to evaluate the expression patterns of these molecules in relationship to the malignant infiltrates, and to directly assess the degree and pattern of microvessel density in the marrow.

The purpose of this study was to further investigate the role of angiogenesis in CLL by evaluating the topography and extent of angiogenesis in a group of CLL bone marrow biopsies, in particular the relationship of microvessels to the infiltrates. We also wanted to study the expression of pro and antiangiogenic vascular factors in CLL cells to more precisely document the cell types producing these factors. To evaluate the role, if any, of localized hypoxia in upregulation of angiogenesis in CLL, we also examined the expression of the VEGF transcriptional regulator, HIF-1α. Finally, in a subset of cases we correlated our findings with genetic and phenotypic factors that have prognostic significance in CLL.

## Materials and Methods

### Institutional review board

The following experiments were performed with the approval of the Institutional Review Board of Northwestern University Feinberg School of Medicine and the Mayo Clinic.

### Cases

We studied 42 adult patients with typical clinical, flow cytometric, and morphologic evidence of CLL [16]. For the purposes of this study we defined two groups.

#### Group 1 – CLL marrow immunohistochemical analysis

We retrospectively selected 28 bone marrow core biopsies from 21 patients with a diagnosis of CLL who were also being evaluated for disease status prior to beginning therapy with the antiangiogenic drug thalidomide as part of a North Central Cancer Treatment Group Center (NCCTG) clinical study. Marrow specimens were decalcified and paraffin-embedded sections cut at 5 μm intervals were stained with hematoxylin-eosin for initial morphologic evaluation. Immunohistochemistry was performed on serial sections from the core biopsies using antibodies against CD3 and CD20 (for evaluation of extent of marrow involvement by CLL), CD34 (for evaluation of microvessel density), p53, VEGF, Hif-1α, TSP-1, and bFGF and the VEGF receptors VEGFR-1 (Flt-1) and VEGFR-2 (Flk-1). Manufacturer and clone details are summarized in Table 1. HIF-1α staining was performed using signal amplification. All other immunohistochemistry was performed using direct antibody labeling.

**Table 1:** Antibodies used for immunohistochemistry

**Antibody**	**Manufacturer**	**Class**	**Clone**
CD3	Dako, Glostrup, Denmark	IgG	Polyclonal
CD20	Dako, Glostrup, Denmark	IgG2a	L26
CD34	Dako, Glostrup, Denmark	IgG1	QBend 10
p53	Dako, Glostrup, Denmark	IgG2b	D0–7
VEGF	Santa Cruz, Santa Cruz, CA	IgG	Polyclonal
HIF-1α	Novus, Littleton, CO	IgG2b	H1α67
TSP-1	NeoMarkers, Fremont, CA	IgG1	A6.1
bFGF	R&D, Minneapolis, MN	IgG2A	10043
VEGF-R1	NeoMarkers, Fremont, CA	IgG	Polyclonal
VEGF-R2	NeoMarkers, Fremont, CA	IgG	Polyclonal

Analysis of microvessel density (MVD) was performed on CD34-stained sections as described previously [5]. Briefly, bone marrow biopsy sections were examined using a model BH-50 Olympus Microscope (Tokyo, Japan) with a 60× objective and a 10× ocular lenses. The number of microvessels per field was measured, and we noted whether a given field was involved by CLL, uninvolved, or partially involved. We also indicated whether a given field included a proliferation center or was at the edge or center of a nodular infiltrate. We compared the average (arithmetic mean) microvessel density of involved versus uninvolved fields in CLL cases and CLL versus normal cases using the student T-test at the 5% confidence level. We also noted the relative microvessel density in proliferation centers versus involved fields not containing proliferation centers and fields containing the edges of nodular infiltrates versus the centers.

In an effort to correlate the results of marrow MVD analysis with other CLL prognostic indicators, additional patient material was used for genetic analysis for other features with known prognostic significance. Fluorescence in situ hybridization (FISH) analysis of patient cells was performed using some commercial FISH probes (Vysis, Downers Grove, IL) and some homebrew FISH probes to detect the presence of two recurring abnormalities predictive of poor prognosis in CLL, trisomy 12 and mutation of chromosome 17p in the vicinity of the p53 locus, and a common genetic marker predictive of a more favorable course, abnormality of chromosome 13q14, using techniques published elsewhere [17,18].

Flow cytometric immunophenotypic analysis of patient blood samples was performed to assess lymphocyte expression of CD38, a poor prognostic indicator in CLL. Lymphocytes from patient samples were separated from the other cellular elements using the ficoll-hypaque gradient technique, and were stained using a commercially available phycoerythrin (PE) labeled anti-CD38 and fluorescein isothiocyanate (FITC)-labeled anti-CD19 antibodies (Becton Dickinson). Two-color analysis was performed using a FACScan flow cytometer manufactured by Becton-Dickinson. A positive result was defined by CD38 expression in ≥ 20% of cells.

RNA extracted from patient bone marrow samples was RT-PCR-amplified and sequence analysis of the cDNA of the IgV_H _region was performed as previously reported [19]. Using a comparison to the V BASE sequence directory using DNAPLOT software, cases with <2% deviation from the most familiar germline Ig sequence were designated "non mutated", the others as "mutated" type B-cell clones.

#### Group 2 – Flow cytometric analysis of CLL B-cells

21 additional cases with peripheral blood and/or bone marrow involvement by CLL encountered in the clinical population at Northwestern Memorial Hospital were selected for analysis of p53, VEGF, HIF-1α, and TSP-1 (cells from Group 1 patients were unavailable for testing for these antigens). Lymphocytes from patient samples were separated from the other cellular elements using the ficoll-hypaque technique. The lymphocytes were then stored at -30°C until analysis. The antibodies were conjugated to fluorescein isothiocyanate (FITC), phycoerytherin (PE), PE-cyanine 5 molecular conjugate (PE-CY5), or phycoerythrin-Texas red molecular conjugate (ECD). Detailed antibody information is summarized in Table 2. To access the cytoplasmic antigens, the cells were treated with Intreprep permeabilization reagents (Beckman Coulter, Miami, FL) as recommended by the manufacturer. Stock solutions of 200 ug/mL of VEGF and TSP-1 were made, and 5 μL were used per sample. The secondary antibody for VEGF was anti rabbit Alexa 488 (Molecular Probes, Eugene, OR). 10 μL were used per test. For Hif-1α, a stock solution of 2.95 mg/mL was made. The solution was diluted 1:10 in phosphate buffered saline, and 3.4 μL were used for each sample. 2 ug of p53 antibody were used per sample. Rat anti-Mouse IgG was used as the secondary antibody for TSP-1 and Hif-1α. 10 μ of anti CD5 and CD19 were used per sample. Four color flow cytometric immunophenotypic analysis was performed using an instrument manufactured by Beckman Coulter.

**Table 2:** Antibodies used for flow cytometry

**Antibody**	**Manufacturer**	**Class**	**Clone**	**Fluorochrome**
CD5	Beckman Coulter, San Diego, CA	IgG2a	BL1a	PC5
CD19	Beckman Coulter, San Diego, CA	IgG1	J4.119	ECD
p53	Dako, Glostrup, Denmark	IgG2b	D0–7	FITC
TSP-1	NeoMarkers, Fremont, CA	IgG1	A6.1	PE
VEGF	Santa Cruz, Santa Cruz, CA	IgG	polyclonal	FITC

Abbreviations: PC5- phycoerythrin-cyanin 5.1; ECD- PE-Texas red; FITC- fluorescein isothiocyanate; PE- phycoerythrin.

To validate the performance of the angiogenesis-related antibodies used in this study, flow cytometric analysis identical to that described for patient and control samples was also performed on cell lines with known expression of the antigens in question. For VEGF, the breast cancer-derived MCF7 cells were used [20]. The human umbilical vein endothelial cell-derived cell line HUVEC was used for TSP-1 [21]. The SV40 (simian virus 40) -transformed cell line HSF4-T12 was used to assess expression of p53 [22].

### Controls

A control group consisting of 10 patients was identified with bone marrow biopsies that were not involved by a malignant process, including CLL. The biopsies were processed, cut, and stained with anti-CD34 in an identical fashion to that described for the cases.

A second control group of 10 bone marrow/peripheral blood cases was selected from the clinical case load of Northwestern Memorial Hospital. All cases had an identifiable benign B lymphocyte population and had no evidence of malignancy. Lymphocytes were separated from the other cellular elements as described above and were analyzed by flow cytometry in an identical fashion to that described for the cases. Whole peripheral blood from two additional cases was analyzed without prior separation of lymphocytes for assessment of granulocyte VEGF expression.

## Results

### Histopathology

Cases of CLL from group 1 had marrow involvement by CLL B-cells ranging from <10% to100% of the marrow cellularity. 6 patients had one or more proliferation/growth centers, which contained cells with slightly larger overall size, slightly dispersed nuclear chromatin, prominent nucleoli, and an increased number of mitotically active cells. Patterns of marrow involvement included diffuse (12 biopsies), interstitial (6 biopsies), nodular (7 biopsies), or a combination of patterns (3 biopsies).

### Microvessel density (MVD)

Compared to a control group of 10 cases, the mean MVD of the 21 group 1 CLL cases was higher than that of the normal control group. The average MVD in the CLL group was 23.8 (range 13.4–35.9) while the average of the control group was 14.6 (range 9.2–22.1). Although there was some overlap in average MVD in the CLL and control groups, the overall difference in mean microvessel density between the CLL and control groups was statistically significant with a p value of 0.0002 using the 2-tailed students T-test.

**Figure 1:**
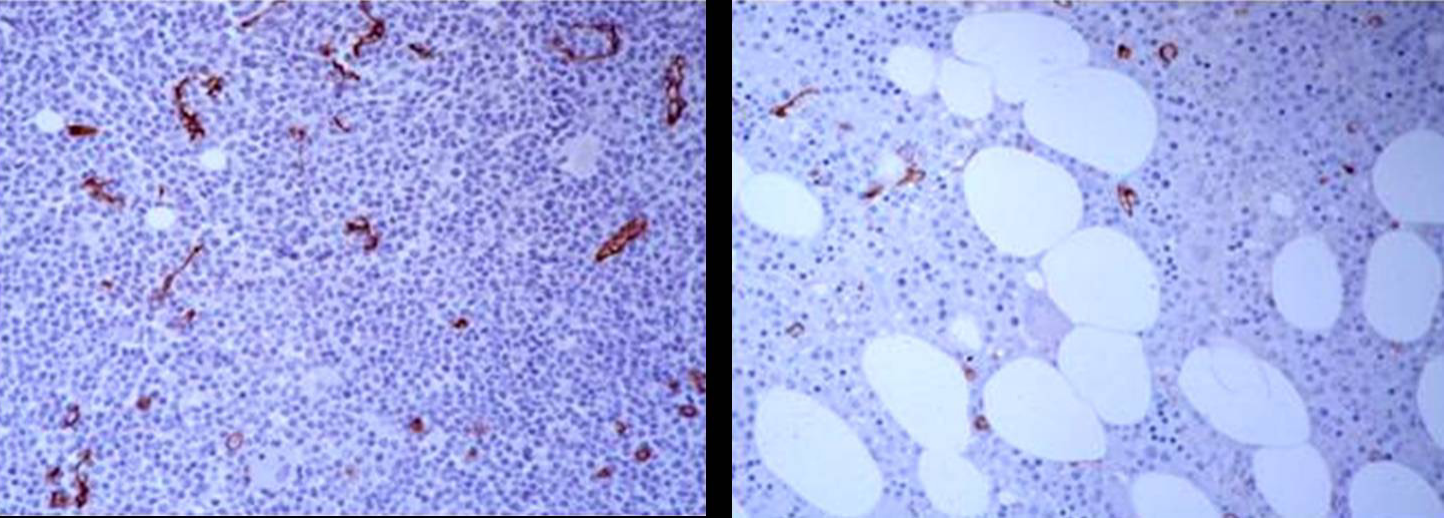
**Increased Microvessel Density in CLL vs Controls**. Microvessel density was highest at the periphery of focal infiltrates. This is a case of CLL (left panel) with nodular lymphoid infiltrates. The image shows an increase in CD34+ microvessels at the leading edge of an infiltrate. In the control tissue (right panel), the microvessel density is somewhat less.

**Figure 2:**
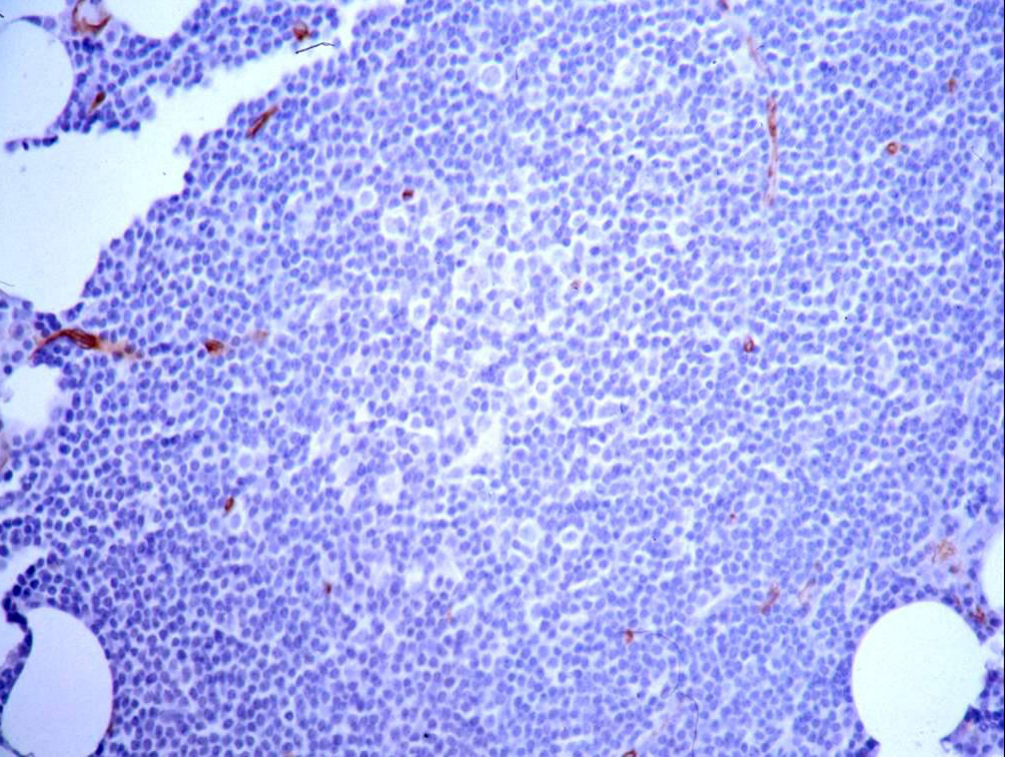
**Microvessel Density is not Enhanced in Proliferation Centers**. A proliferation center is present at the center of the field, which has a lower number of CD34+ microvessels than surrounding areas.

The distribution of microvessels in the CLL biopsies differed somewhat from that in the control patients as the latter had distribution of blood vessels in a uniform fashion throughout the biopsies. The MVD was highest at the periphery of focal infiltrates, as has been described in solid tumors (Figure 1)[23]. In the 6 CLL cases with ≥ 1 uninvolved high power field, the average of the involved fields were slightly higher than the uninvolved fields (24 vs. 21 microvessels per high power field); this difference was not statistically significant. In cases with proliferation centers, MVD was not enhanced within the proliferation centers relative to the rest of the infiltrate, and approximated that of uninvolved areas (Figure 2).

### VEGF

**Figure 3:**
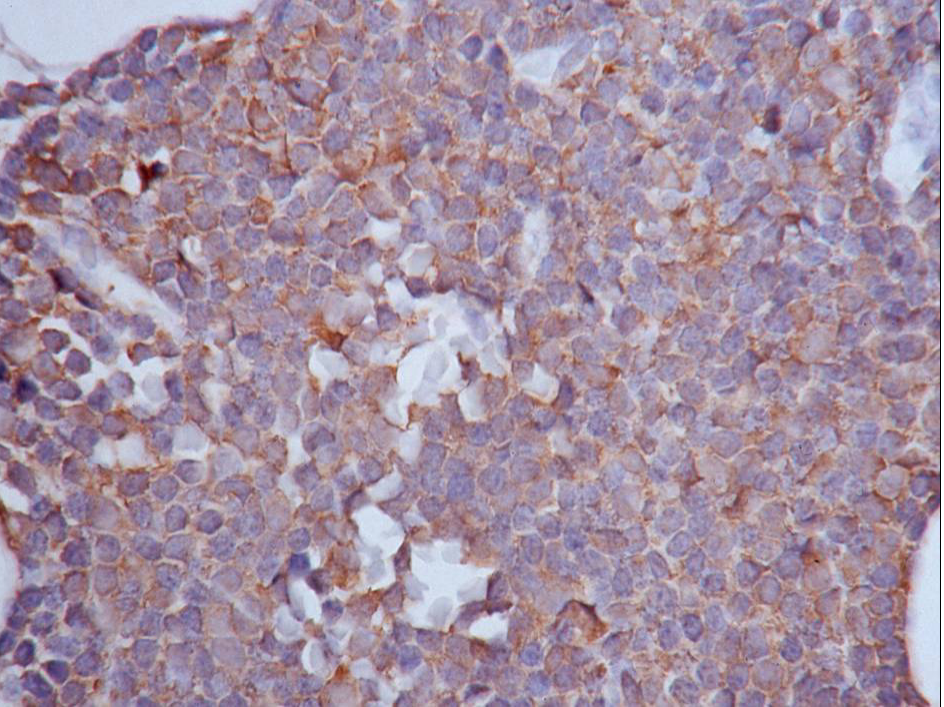
**CLL Cells Express VEGF**. A) CLL Cells in all 21 cases tested by immunohistochemistry were consistently VEGF positive. The majority of the cells in this field are lymphocytes which are VEGF positive. Granulocytes had more intense VEGF reactivity, and erythroid cells, as seen at the center of the field, were negative.

**Figure 4:**
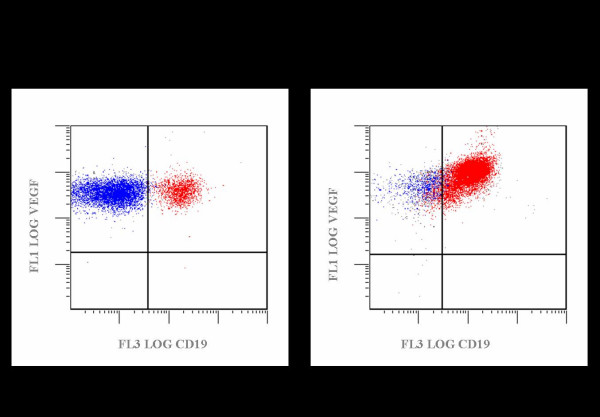
**Bright VEGF Expression is Identified in CLL Cells by Flow Cytometry**. 21 cases were examined by flow cytometry for VEGF expression. The panel on the left is a plot of VEGF vs CD19 expression in a control case. The level of VEGF expression by the CD19+ B cells in the control cases was equal to that of the CD19- T cells. A plot of VEGF vs CD19 in one of the CLL cases is illustrated in the right panel. The CD19+ CLL Cells were 1.4–2.0× Brighter than the CD19-CD5+ T cells in the same samples. **VEGFR-2 (KDR, Flk-1)**: In most cases, only scattered cells in the infiltrates were positive, with the exception of two cases, one of which is illustrated, in which the vast majority of lymphocytes were positive. The microvessel density of these two cases did not markedly differ from the other cases in this group.

CLL cells in all 21 cases tested by immunohistochemistry were consistently VEGF positive (Figure 3). Marrow, which could be easily distinguished from the marrow lymphocytes, had more intense VEGF reactivity, and erythroid precursor cells were negative. 21 cases were examined by flow cytometry for VEGF expression (Figure 4). The level of VEGF expression by the CD19+ B cells in the control cases was equal to that of the CD19- T cells. A plot of VEGF versus CD19 in one of the CLL cases is illustrated in the right panel of Figure 3. The CD19+ CLL cells were 1.4–2.0× brighter for VEGF than the CD19-CD5+ T cells in the same samples.

### VEGFR-1 (Flt-1)

**Figure 5:**
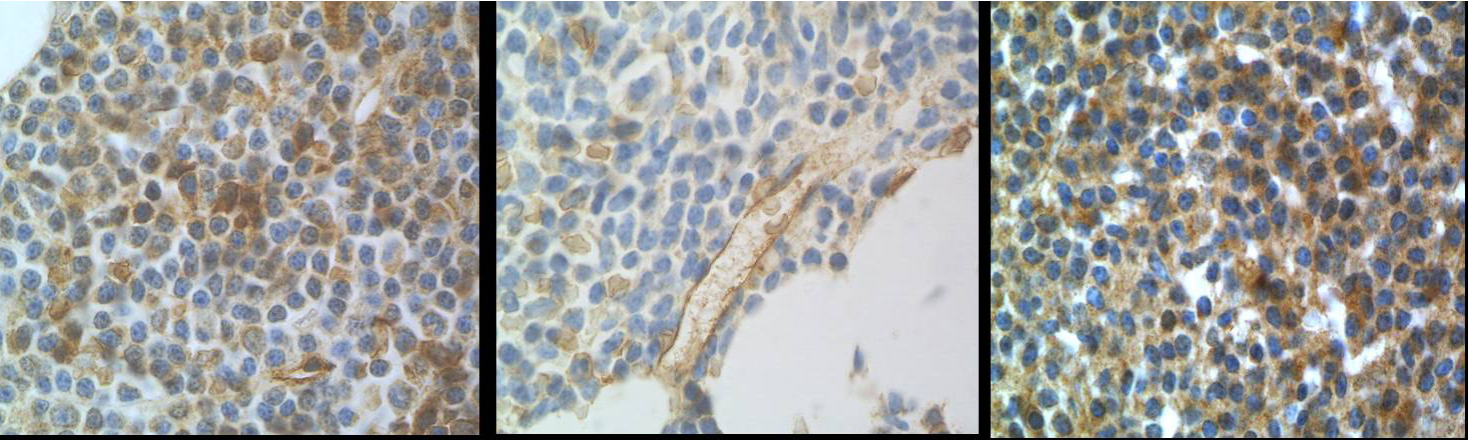
**VEGF receptors. VEGFR-1 (Flt-1)**. Lymphocyte staining was variable and highlighted only a minority of cells in most cases. In the case illustrated in the left panel, many cells in the infiltrate were positive. Very few positive cells were present in the case illustrated in the middle panel. In all cases, granulocytes demonstrated intense cytoplasmic reactivity, scattered erythroid precursors had a predominantly membranous pattern of staining, and megakaryocytes had faint cytoplasmic reactivity. Endothelial cells, which could be identified in most cases, were positive and served as useful internal positive controls. **VEGFR-2 (KDR, Flk-1)**: In most cases, only scattered cells in the infiltrates were positive, with the exception of two cases, one of which is illustrated in the right panel, in which the vast majority of lymphocytes were positive. The microvessel density of these two cases did not markedly differ from the other cases in this group.

The 21 group 1 cases were evaluated for VEGFR-1 expression by immunohistochemistry (Figure 5). In all cases, granulocytes demonstrated intense cytoplasmic reactivity, scattered erythroid precursors had a predominantly membranous pattern of staining, and megakaryocytes had faint cytoplasmic reactivity. Endothelial cells, which could be identified in most cases, were positive and served as useful internal positive controls. Lymphocyte staining for VEGFR-1 was variable and highlighted only a minority of cells in most cases.

MVD was highest at the periphery of focal infiltrates, was not enhanced in proliferation centers, and was increased irrespective of the presence or absence of cytogenetic/immunophenotypic markers of aggressivity. By IHC, CLL cells were VEGF(+), HIF-1a (+), TSP-1(-), VEGFR-1(+), and VEGFR-2(+). By FC, CLL cells were 1.4–2.0-fold brighter for VEGF than T cells and were TSP-1(-). CLL demonstrates enhanced angiogenesis, with increased MVD, upregulated VEGF and downregulated TSP-1. Upregulation of HIF-1a in all CLL cases suggests localized tissue hypoxia as an important stimulant of microvessel proliferation. The presence of VEGF receptors on CLL cells implies an autocrine effect for VEGF. Differences in MVD did not correlate with traditional genetic/immunophenotypic markers of aggressivity.

### VEGFR-2 (KDR, Flk-1)

In the 21 group 1 cases, a differential pattern of staining was identified (Figure 5). Granulocytes demonstrated the most intense reactivity. Erythroid precursors and megakaryocytes had a weaker pattern of reactivity compared to granulocytes that did not appear distinctly membranous. In most cases, only scattered cells in the infiltrates were positive, with the exception of two cases in which the vast majority of lymphocytes were positive. The MVD of these two cases did not markedly differ from the other cases in this group.

### HIF-1 α

**Figure 6:**
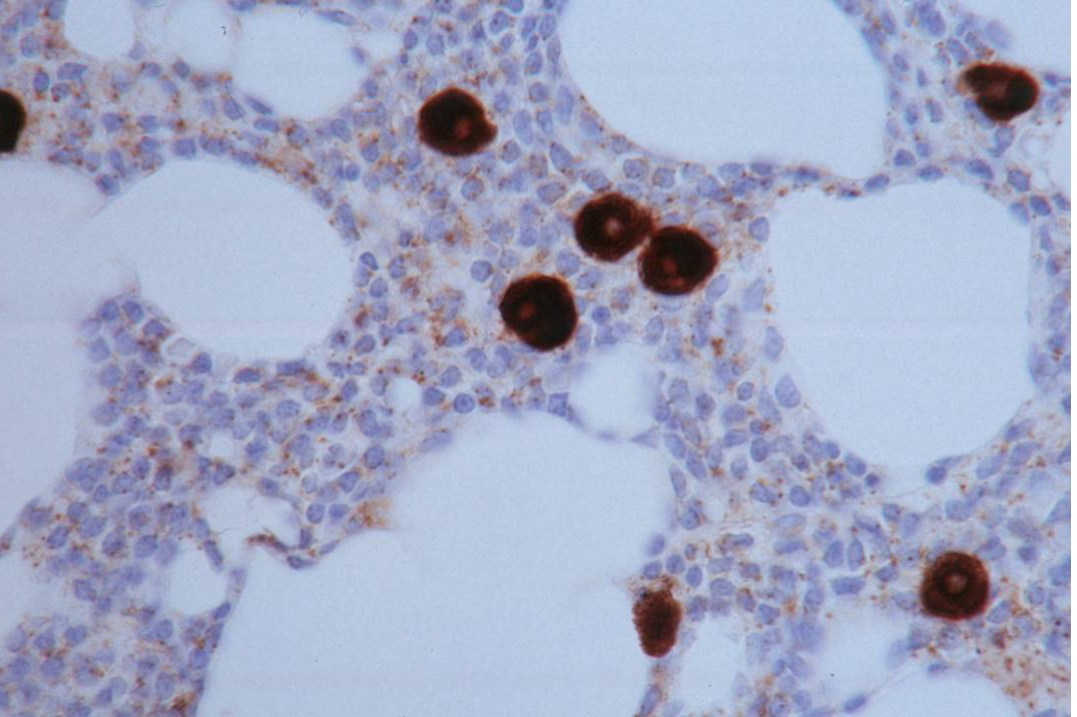
**Hif-1 α Expression by CLL cells**. The 21 group 1 cases were evaluated for Hif-1 α expression. Lymphocytes from all cases were positive.

The 21 group 1 cases were evaluated for HIF-1 α expression. Lymphocytes from all cases were positive. Other marrow hematopoeitic elements were negative, with the exception of mast cells (Figure 6).

### TSP-1

**Figure 7:**
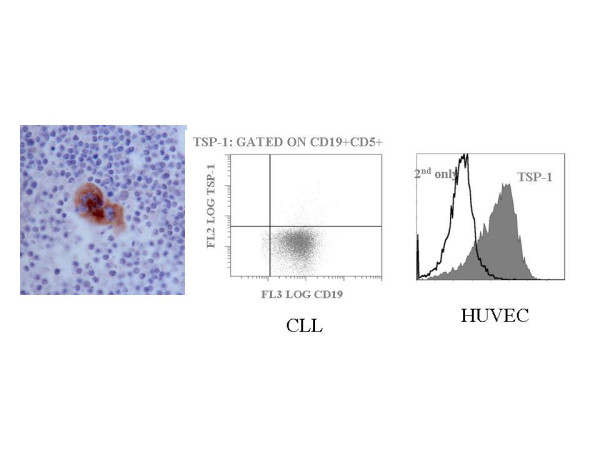
**TSP-1 is not expressed by CLL cells**. CLL cells in all cases tested for TSP-1 expression by immunohistochemistry were negative. In the left panel is a case of CLL with a field of TSP-1 negative lymphocytes. At the center of this field is an internal positive control, a megakaryocyte. The TSP-negativity of CLL cells was verified by flow cytometry. All 21 cases tested had results similar to the cases illustrated in the middle panel. HUVEC cells, which are known to express TSP-1, were positive by flow cytometry (right panel).

CLL cells in all cases tested for TSP-1 expression by IHC were negative. Megakaryocytes were useful positive internal controls (Figure 7). The uniform TSP-1 negativity of CLL cells was also noted using flow cytometry. All 21 cases tested had similar results. In contrast, HUVEC cells, which are known to express TSP-1, were positive by flow cytometry (Figure 7).

### bFGF

A differential pattern of staining was noted in the 21 group one cases. The granulocytes were intensely positive, the lymphocytes had fainter staining, and erythroid precursors were negative.

### P53

In two cases, the majority of lymphocytes in the infiltrates were positive. The other nineteen cases were negative or demonstrated only rare scattered positive cells.

### Interphase cytogenetics

Correlation with genetic and immunophenotypic prognostic factors was performed on18 Group 1 cases, using probes as described elsewhere [18].

FISH was performed for analysis using two abnormalities predictive of poor prognosis, trisomy 12 (D12Z3 for chromosome 12 centromere and MDM2 for 12q15) and abnormalities of chromosome 17 (p53at 17p13.1 and D17Z1 for the chromosome 17 centromere), and a marker predictive of a more favorable course, abnormality of chromosome 13q (D13S319 at 13q14 and LAMP1 at 13q34). Cases with trisomy 12 (2 cases) or chromosome 17 abnormalities (1 case) had a similar mean MVD to 15 cases with partial loss of chromosome 13q (13 cases) or no demonstrable abnormalities using these probes (2 cases). The case with abnormal chromosome 17 also had increased p53 staining by immunohistochemistry. No cases had evidence of CCND1/IgH [t(11;14)] fusion [18].

### CD38 and/or IgVH analysis

15 Group 1 cases were tested for CD38 expression and or IgV_H _gene mutation. Eight cases with >20% of the CLL cells expressing CD38 and/or IgV_H _gene in germline configuation, both of which are poor indicators, had an average MVD that was similar to 7 cases that were both CD38 negative and had mutated IgV_H_.

## Discussion

CLL is a complex disease characterized by a progressive accumulation of CD5+ B-lymphocytes in the peripheral blood, bone marrow, lymph nodes, and other sites. Another important host system for the survival of the malignant population is the vasculature of the bone marrow and other involved sites. We report the results of a series of experiments performed on bone marrow trephine biopsies and bone marrow/peripheral blood samples from patients with CLL that support earlier observations that dysregulated angiogenesis is a common phenomenon in this disease. The proangiogenic factors VEGF and HIF-1α are expressed by the malignant cells and are associated with increased microvessel production in the bone marrow milieu. The potent antiangiogenic factor TSP-1 is not produced by the malignant cells in the marrow. In addition to endothelial surfaces, VEGFR-1 and VEGFR-2 are also expressed on the CLL cells, implying that VEGF may act both on the normal endothelial cells and on the malignant population.

Micovessel density is increased throughout. Several observations suggest a relatively localized paracrine effect of proangiogenic factors in CLL bone marrows and the possible importance of angiogenesis in the metabolically active edges of CLL infiltrates. Although most cases had a diffuse growth pattern, in cases with a nodular pattern of growth, the edges of the nodules had a relatively higher microvessel density than the centers or uninvolved areas of marrow.

Observations such as these are also noted in other malignancies, including both hematologic neoplasms and solid tumors. The explanation for this lies in the production of proangiogenic growth factors and hormones by tumor cells and/or the nontumoral matrix [24-26]. In addition, the production of growth factors in the tumor cells is often controlled by transcriptional regulators, the most prominent of which appears to be HIF-1α.

A major proangiogenic factor is VEGF, a homodimeric glycoprotein (molecular weight ~45 kD) encoded by a gene on chromosome 6p21-p12 [27,28] which stimulates angiogenesis and vascular permeability by interacting with the tyrosine kinase receptor-2 (VEGFR-2 or KDR/Flk-1) and -1 (VEGFR-1 or Flt-1) [29]. VEGF expression was apparent in both the granulocytes and lymphocytes of the CLL cases. Although there may be a slight increase in microvessel density in areas of involvement, there is an overall increase in microvessel density thoughout. The mere production of proangiogenic factors is not sufficient to explain the differential MVD noted in our patients. Another possible explanation for the localized microvessel production in CLL is the differential expression of other pro- and antiangiogenic factors such as HIF-1α and TSP-1 that may act in concert with VEGF to induce neovascularization.

VEGF is a mitogen that presumably could have activity in any cells expressing the VEGF receptors VEGFR-1 (Flt-1) and VEGFR-2 (Flk-1), and co-expression of at least one of its receptors supports the theory of an autocrine role for the cytokine in addition to the paracrine role associated with angiogenesis [30-32]. Knowledge of the extent of VEGF receptor expression in normal tissues is currently limited, but VEGFR-2 appears to demonstrate lineage restriction that is limited to endothelial cells in normal conditions [33], and is expressed in certain leukemias [34]. In CLL, we have noted that both receptor classes are expressed on the malignant cells, and Kay et al have shown that mRNA encoding the VEGF receptors is upregulated in CLL cells [12]. In the model proposed by Kay et al VEGF acts autocrinously, since two different classes of VEGF receptors are expressed on CLL cells. We provide further evidence of this by demonstrating the presence of VEGFR-1 and VEGFR-2 immunohistochemically in the malignant cells. In this regard, VEGF could act like the receptors for the growth factors bFGF and platelet-derived growth factor (PDGF), which are expressed in a wide variety of cell types and have a broad spectrum of mitogenic activity [28]. Interestingly, there was a broad range of VEGF receptor expression in the lymphocytes. In some cases, large numbers of cells expressed VEGF receptors and in others receptor expression was virtually absent. We did not find a clear association of this finding with stage or MVD; however this may represent evidence of heterogeneous acquisition of an autocrine phenotype by CLL cells.

In normal systems, expression of the various angiogenesis-related factors is regulated, resulting in a dynamic equilibrium in which pro-and antiangiogenic factors are balanced. In conditions of localized tissue hypoxia, VEGF expression is enhanced by the transcription regulator HIF-1α. Increased expression of HIF-1α mRNA was noted by Kay et al in CLL cells grown in vitro [12]. In our cases, a speckled pattern of staining with antibody directed against HIF-1 α was consistently present in CLL cells. Other cells were negative, with the exception of mast cells, which have a proven role in angiogenesis [35,36], although production of HIF-1α by mast cells has not previously been reported. Because granulocyte staining for HIF-1α in our specimens was not observed, it seems unlikely that the cytoplasmic mast cell staining observed in our patients is the result of nonspecific (antibody-independent) interactions. The pattern of lymphocyte staining with this antibody suggests a tight clustering of antigens within the CLL cytoplasm, the significance of which is currently unknown.

In the smaller number of cases we have tested for the FISH detectable abnormalities, immunoglobulin mutational status, and immunophenotypic parameters predictive of poor prognosis, however, we found no difference in MVD between those patients with one or more of these poor prognostic indicators and their angiogenic status as determined by MVD.

In contrast to the apparent lack of correlation of these prognostic indicators with angiogenic status, Kini et al have identified that degree of angiogenesis, using MVD as an index, correlated with stage [5], and Molica et al have demonstrated that MVD at diagnosis correlates with upstaging and progression-free survival [37]. These findings suggest that dysregulation of angiogensis is common in CLL, and as such may represent an early event in leukemogenesis. Since the clinical course of CLL may last many years, the acquisition of other genetic mutations may augment the earlier dysregulation of angiogenesis, accounting for the increased MVD seen in higher stage individuals. Patients with a shorter duration of disease may have more modest changes in angiogenic status.

## Authors' contributions

JLF carried out flow cytometry and immunohistochemistry experiments and drafted the manuscript. NEK, CLG, and SEC participated in the design of the study and reviewed the manuscript. GWD carried out cytogenetic studies. LCP drafted the manuscript.
